# Direct costs of common osteoporotic fractures (Hip, Vertebral and Forearm) in Iran

**DOI:** 10.1186/s12891-021-04535-8

**Published:** 2021-07-31

**Authors:** Marziyeh Rajabi, Afshin Ostovar, Ali Akbari Sari, Sayed Mahmoud Sajjadi-Jazi, Noushin Fahimfar, Bagher Larijani, Rajabali Daroudi

**Affiliations:** 1 Department of Health Management and Economics, School of Public Health, Tehran University of Medical Sciences, Poursina Ave, Tehran, Iran; 2 Osteoporosis Research Center, Endocrinology and Metabolism Clinical Sciences Institute, Tehran University of Medical Sciences, No.10-Jalal–e-Ale-Ahmad St, Chamran Hwy, P. O. Box: 14117-13119, Tehran, Iran; 3 Endocrinology and Metabolism Research Center, Endocrinology and Metabolism Clinical Sciences Institute, Tehran University of Medical Sciences, Tehran, Iran; 4 Cell Therapy and Regenerative Medicine Research Center, Endocrinology and Metabolism Molecular-Cellular Sciences Institute, Tehran University of Medical Sciences, Tehran, Iran

**Keywords:** Costs, Fracture, Osteoporosis, Quality of life

## Abstract

**Background:**

Osteoporotic fractures impose significant costs on society. The objective of this study was to estimate the direct costs of the hip, vertebral, and forearm fractures in the first year after fracture incidence in Iran.

**Methods:**

We surveyed a sample of 300 patients aged over 50 years with osteoporotic fractures (hip, vertebral, and forearm) admitted to four hospitals affiliated to Tehran University of Medical Sciences, Iran, during 2017 and were alive six months after the fracture. Inpatient cost data were obtained from the hospital patient records. Using a questionnaire, the data regarding outpatient costs were collected through a phone interview with patients at least six months after the fracture incidence. Direct medical and non-medical costs were estimated from a societal perspective. All costs were converted to the US dollar using the average exchange rate in 2017 (1USD = IRR 34,214)

**Results:**

The mean ± standard deviation (SD) age of the patient was 69.83 ± 11.25 years, and 68% were female. One hundred and seventeen (39%) patients had hip fractures, 56 (18.67%) patients had vertebral fractures, and 127 (42.33%) ones had forearm fractures. The mean direct cost (medical and non-medical) during the year after hip, vertebral and forearm fractures were estimated at USD5,381, USD2,981, and USD1,209, respectively.

**Conclusion:**

The direct cost of osteoporotic fracture in Iran is high. Our findings might be useful for the economic evaluation of preventive and treatment interventions for osteoporotic fractures as well as estimating the economic burden of osteoporotic fractures in Iran.

**Supplementary Information:**

The online version contains supplementary material available at 10.1186/s12891-021-04535-8.

## Introduction

Osteoporosis is a major public health problem through associated fragility fractures [1]. The worldwide prevalence of osteoporosis is estimated to be over 200 million and it contributes to about 9 million fractures each year [2]. In the United States, the prevalence of osteoporosis in people over the age of 50 was estimated to be about 10.3 percent (10.2 million people) in 2010, and over 1.5 million fractures per year are attributed to osteoporosis [3, 4]. Based on a systematic review and meta-analysis study, the prevalence of osteoporosis was estimated at 17% (95% CI [13%, 20%]) in Iran [5].

The clinical significance of osteoporosis lies in the resulting fractures. The most common fractures caused by osteoporosis include hip, spinal, and forearm fractures [6]. The global incidence of hip fracture was estimated to be 2.7 million cases in 2010 [7]. The findings of a systematic literature review of hip fracture incidence studies showed that the age-standardized annual incidence of hip fractures in women (per 100,000) was between 2 in Nigeria and 574 in Denmark [8]. The incidence of hip fractures in women in Iran is estimated at about 400/per 100,000 people [2]. The number of hip fractures was estimated to be 50,000 cases in 2006, and according to predictions, it will reach 62,000 cases in 2020 [9]. Data on the incidence of other osteoporotic fractures are lacking in most of the countries in the world, especially in the low and middle-income countries [10, 11].

Osteoporosis and its related fractures, in addition to their effects on people's quality of life, impose a significant economic burden on families and the health system [1]. Financial costs, reduced quality of life, and premature death are among the consequences of osteoporosis and osteoporotic fractures [1, 4]. In the United States, the direct cost of osteoporosis in 2005 was estimated to be between $ 13.7 billion and $ 20.3 billion and according to forecasts, by 2025, more than 3 million osteoporosis fractures will occur annually, costing $ 25.3 billion [12, 13]. In 2005 in China, the direct cost per patient with a hip fracture was estimated to be $ 3,600. The cost of treating hip fractures in 2005 was about $ 1.5 billion and is estimated to increase to $ 12.5 billion by 2020 [14]. In 2009, the direct medical costs of hip fractures in Iran was estimated to be $ 28 million, and it is predicted to increase to $ 250 million by 2050 [9].

To the best of our knowledge, there is limited knowledge about the economic burden of osteoporosis and fragility fractures in Iran [15]. Therefore, this study aimed at calculating the direct medical and non-medical costs of common osteoporotic fractures during one year after the fracture in Iran, in total and by fracture type.

## Methods

This was a cross-sectional descriptive study in which we calculated the direct medical and non-medical costs imposed on patients within one year after the common osteoporotic fractures.

Direct medical costs included costs of hospitalization, medications, diagnostic tests, physician visits, and diagnostic imaging. Direct non-medical costs included costs of travel for treatment, absenteeism, Informal Care and services, and Patient Time Cost.

### Study participants

The study samples included patients visiting teaching hospitals affiliated to Tehran University of Medical Sciences (TUMS) in 2017 due to common fractures caused by osteoporosis, including hip, vertebral, and forearm fractures. Inclusion criteria were age ≥ 50 years, the incident of hip, vertebral or forearm fragility fractures (based on ICD_10 code) in 2017, T-score ≤ -2.5 standard deviation (SD) or fracture due to minor trauma, admission in four teaching hospitals affiliated to TUMS (including Shariati, Sina, Baharlo, Ziaeian hospitals), and surviving at least six months after the fracture incidence. The total number of patients was 476, of whom 71 died, and another 105 were excluded due to unwillingness to participate in the study or incorrect telephone number. Finally, 300 patients participated in the study.

### Data collection

Inpatient costs were extracted from hospital records. Outpatient costs were also collected using a questionnaire through telephone interviews with patients. Since outpatient costs occur after hospital discharge, interviews with patients were performed at least 6 months after the occurrence of fracture. In the case of patients unable to answer questions, a family member or caregiver was interviewed who had sufficient information.

The study questionnaire consisted of 4 main sections. The first section of the questionnaire was related to patients' demographic information, including age, gender, marital status, place of residence, education status, employment status, and health insurance coverage status. The second section of the questionnaire was related to clinical information and a history of osteoporosis and osteoporotic fractures. In the third section, questions related to resource consumption were asked to calculate direct medical expenses. In this part of the questionnaire, patients were asked what services they had received over the past six months due to osteoporosis and related fractures, as well as the number of times each service was used.

For this purpose, a list of services including physician visits, imaging services, medications, diagnostic tests, and rehabilitation services was provided, and patients were asked about the use of each service. The fourth section of the questionnaire was related to direct non-medical costs, and patients were asked about the number of trips, costs of each trip, costs of absenteeism, home care, complementary treatment expenses (such as supplementations), and cost of using equipment such as wheelchairs, cane, etc.

The questionnaire was designed based on previous studies and its validity was confirmed through interviews with clinicians and health economists. Also, a pilot study was conducted and the questionnaire was revised based on its result accordingly.

Details of methods used for calculating direct medical and non-medical expenses by cost items are presented in supplement Table S1.

### Data analysis

Descriptive statistics, including mean, median, and standard deviation, were used to describe the data. To calculate the cost of services for each patient, the number of times each service was used was multiplied by the average price of that service. The price of services was extracted from the official price list for medical services in Iran, approved by the Supreme Insurance Council. Finally, the average annual cost per patient within one year after the fracture had occurred was calculated by the type of cost. Data were analyzed using Microsoft Excel 2013 and Stata software programs (Stata Corporation, College Station, TX). All costs were converted to the US dollar using the average exchange rate in 2017 (1USD = IRR 34,214).

## Results

Overall, 300 patients (65% female) with a mean (SD) age of 69.83 (11.25) were included in the study with a total of 127 (42.3%), 117 (39%), and 56 (17.3%) forearm, hip and clinical vertebral fractures, respectively. Other characteristics are shown in Table 1.

**Table 1:** Sociodemographic characteristics of the participants

Variables	**Type of fracture**							
	**Hip**		**Vertebra**		**Forearm**		**Total**	
	n	%	n	%	n	%	n	%
**Gender**								
Female	76	64.96	34	60.71	94	74.02	204	68.00
Male	41	35.04	22	39.29	33	25.98	96	32.00
**marital status**								
Single	3	2.56	2	3.57	1	0.79	6	2.00
Married	53	45.3	34	60.72	79	62.2	166	55.33
Widow/divorced	61	52.14	20	35.71	47	37.01	128	42.67
**employment status**								
Employment	6	5.13	2	3.57	9	7.09	17	5.67
Housekeeper	57	48.72	26	46.44	82	64.57	165	55.00
No employment	15	12.82	8	14.28	13	10.24	36	12.00
Retired	39	33.33	20	35.71	23	18.11	82	27.33
**Having basic health insurance**								
Yes	116	99.15	50	89.29	119	93.70	285	95.00
No	1	0.85	6	10.71	8	6.30	15	5.00
	**Mean**	**SD**	**Mean**	**SD**	**Mean**	**SD**	**Mean**	**SD**
**Age**	73.58	11.33	69.70	10.39	66.42	10.50	69.83	11.25
**Years of education**	6.44	5.87	6.28	5.63	5.61	5.15	6.06	5.53

**Fig. 1:**
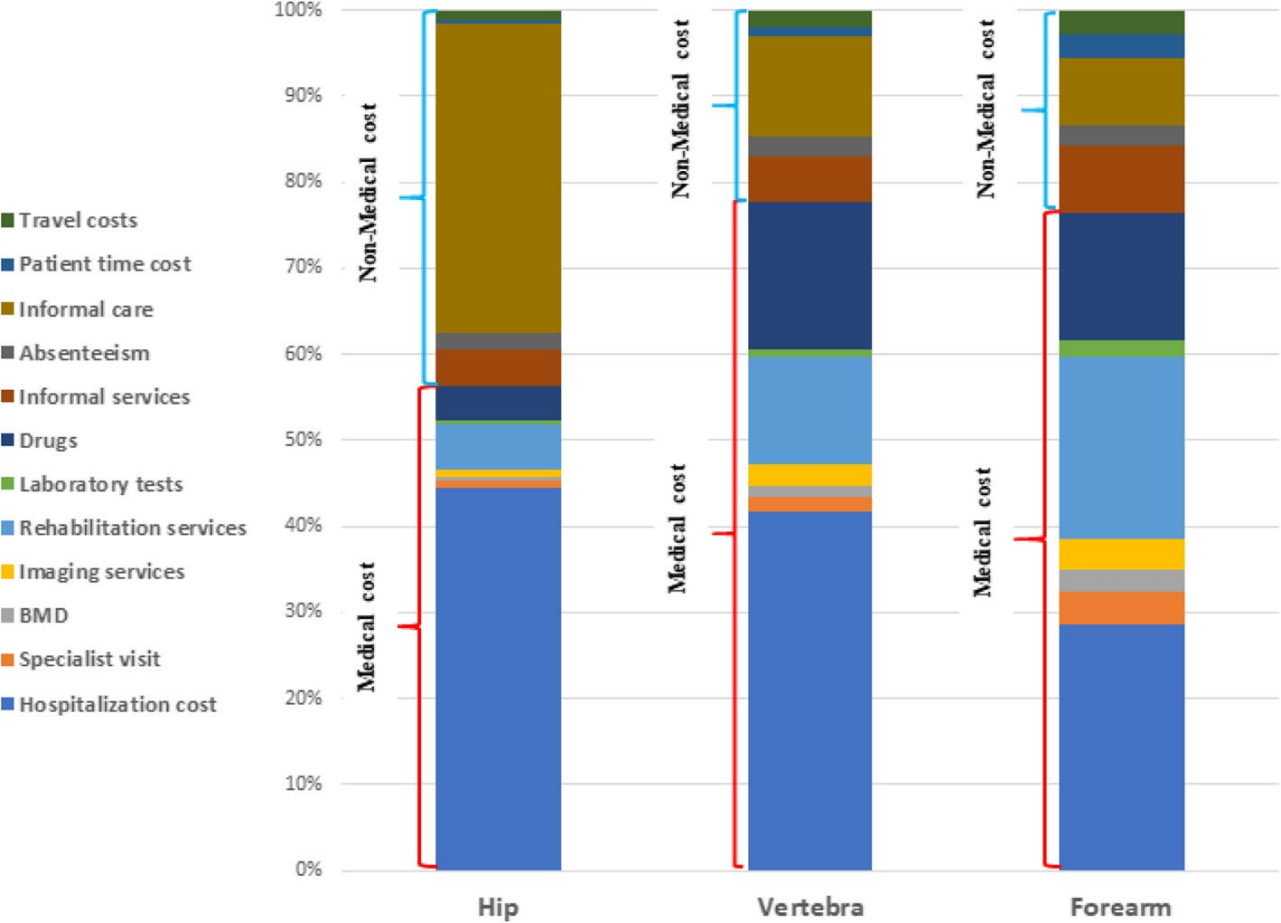
Percentages of direct medical and non-medical costs of osteoporotic fracture by fracture type; BMD: *Bone Mineral Density test*

The average direct medical costs during the first year after hip, clinical vertebral, and forearm fracture were estimated to be $3,029.67, $2,316.59, and $924.90, respectively. In all three groups, inpatient costs accounted for the largest share of direct medical costs. The share of inpatient costs out of the total direct medical costs in the hip, vertebral, and forearm fracture patients were 78.88%, 53.70%, and 37.29%, respectively (Fig. 1). Rehabilitation and medication costs were in subsequent rankings (see Table 2).

**Table 2:** Direct medical cost of osteoporotic fractures (per patient) during the year after the fracture

Cost Item	Type of fracture					
	Hip		Vertebra		Forearm	
	Mean cost(US$)	% of total	Mean cost(US$)	% of total	Mean cost(US$)	% of total
Hospitalization cost	2,389.65	78.88	1,243.93	53.70	344.88	37.29
Specialist visit	45.85	1.51	49.41	2.13	47.56	5.14
BMD^a^ test	25.88	0.85	41.10	1.77	31.49	3.40
Imaging services^b^	50.13	1.65	72.26	3.12	42.22	4.56
Rehabilitation services	282.49	9.32	371.24	16.03	254.82	27.55
Laboratory tests	24.55	0.81	26.86	1.16	25.19	2.72
Drugs^c^	211.11	6.97	511.81	22.09	178.75	19.33
Total	3,029.67	100.00	2,316.59	100.00	924.90	100.00

^a^Bone Mineral Density; ^b^ (MRI, CT-SCAN); ^c^ (osteoporosis drug and painkillers)

Direct non-medical costs are presented separately by the type of fracture in Table 3. The average direct non-medical costs per patient in the hip, clinical vertebral, and forearm fracture were estimated to be $ 2,351.63, $ 664.18, and $ 284.40, respectively. In hip fractures, about 82% of direct non-medical costs were related to patient informal care (see Fig. 1).

**Table 3:** Direct non-medical cost of osteoporotic fractures (per patient) during the year after the fracture

Cost Item	**Type of fracture**					
	**Hip**		**Vertebra**		**Forearm**	
	Mean cost(US$)	% of total	Mean cost(US$)	% of total	Mean cost(US$)	% of total
Informal services (including supplements, Walker, cane, and …)	224.38	9.54	160.42	24.15	95.22	33.48
Absenteeism ( by patient and family members)	109.22	4.64	69.37	10.44	26.35	9.27
Informal care	1,929.02	82.03	344.52	51.87	96.45	33.91
Patient time cost	30.54	1.30	31.41	4.73	31.31	11.01
Travel costs	58.46	2.49	58.46	8.80	35.07	12.33
**Total**	2,351.63	100.00	664.18	100.00	284.40	100.00

The average direct costs (medical and non-medical) during the first year after the fracture were estimated to be $ 5,381.30, $ 2,980.78, and $ 1,209.30 for hip, clinical vertebral, and forearm fractures, respectively (see Table 4). The shares of direct medical costs out of the total direct costs in the hip, clinical vertebral, and forearm fractures were 56.30%, 77.72%, and 76.48%, respectively (see Fig. 1).

**Table 4:** Direct cost (medical and non-medical) of osteoporotic fractures (per patient) during the year after the fracture

Cost Item	Type of fracture					
	Hip		Vertebra		Forearm	
	Mean cost(US$)	% of total	Mean cost(US$)	% of total	Mean cost(US$)	% of total
Direct medical cost	3,029.67	56.30	2,316.59	77.72	924.90	76.48
Direct non-medical cost	2,351.63	43.70	664.18	22.28	284.40	23.52
Total cost	5,381.30	100.00	2,980.78	100.00	1,209.30	100.00

## Discussion

In this study, direct medical and non-medical costs of common fractures caused by osteoporosis, including hip, clinical vertebral, and forearm fractures, were calculated in 2017. The average direct medical costs during the first year after a hip, vertebral, and forearm fracture were estimated to be $ 3,030, $ 2,317, and $ 925, respectively. The average direct non-medical costs per patient in the hip, vertebral, and forearm fractures were estimated to be $ 2,352, $ 664, and $ 284, respectively.

One of the most common osteoporotic fractures is the hip fracture, which impose heavy economic and social burdens on families and communities. It has been estimated that 8–36% of people with hip fractures die within a year of the fracture incidence [16], and 20–30% of these deaths are directly attributed to hip fractures [17]. Only 40–60% of patients return to pre-fracture mobility, and 20–60% of patients who perform personal activities such as washing and dressing without assistance before the fracture are unable to do so alone for more than one year after the fracture. In high-income countries, between 10–20% of patients require long-term care after a hip fracture [18, 19].

Almost all patients with hip fractures are hospitalized. Their length of stay in the hospital is relatively long, and most of them need home care after hospital discharge [18, 20]. The total cost estimated in our study for hip fracture was $ 5,381. According to a systematic review, the average hospital cost per patient with hip fracture in the United States was between $ 8,358 and $ 32,195, with the highest hospital cost among osteoporotic fractures [21]. The results of a more recent study in the United States also showed that the average length of hospital stay for a patient with hip fracture was 5.6 days and the average hospital cost was $14,744 [22]. The results of a study in the UK demonstrated that the average hospital costs for hip fractures during the first and second years after fracture were £14,163 and £2,139, respectively [23]. According to a systematic review and meta-analysis conducted by Williamson et al., the average hospital cost for a hip fracture was $10,075 (95% CI [$ 8,322, $ 11,828]) globally [24].

One study conducted by Wiktorowicz et al. revealed that primary hospital costs account for about one-third of total patient medical costs during the first year after a hip fracture, and about 70% of the costs during the first year are related to informal care, as well as rehabilitation and other outpatient services [25]. According to the above study, the average health and social cost of a hip fracture during the first year after the fracture was $ 43,669 per patient. The average cost of hospitalization per patient was $ 13,331, accounting for about 74% of total medical care costs. Rehabilitation and medication costs, with about 35% and 8%, respectively, were in the next ranks [24]. In our study, hospital costs accounted for about 79% of direct medical costs of hip fractures, and rehabilitation and medication costs were 9% and 7%, respectively. A comparison of our findings with that of others suggest that the share of rehabilitation service costs in our study was lower than in other studies. one reason for this could be the low coverage of rehabilitation services by health insurance in Iran and less use of these services by patients.

Mohd-Tahir et al. conducted a review study in 2017 to estimate the costs of osteoporotic hip fractures in Asian countries. The results of their research showed that few studies had been published on the costs of hip fractures in Asian countries. Only 15 studies had met the criteria for entering in the study. Studies also differed in terms of methodology and type of costs estimated; and most studies have only estimated direct medical costs. According to the so called study, the average cost of a hip fracture in Asia ranged from $ 774 to $ 14,199, with a median cost of $ 2,944 [26]. In our study, the average direct cost of a hip fracture was estimated to be $ 3,030, which is close to the estimated median in Asian countries.

Vertebral fractures are another common osteoporotic fracture that, like hip fractures, increase the risk of death and impose high costs on patients [27]. Ong et al. conducted a systematic review to evaluate the characteristics and outcomes of hospitalized patients due to vertebral fractures. Their study results showed that the rate of hospital admissions due to vertebral fractures in different countries was between 2.8 to 19.3 per 10,000 people per year. The ratio of women to men in different studies was between 57 and 84% with the average of 65% in all studies. Between 20 and 27% of patients died within the first year after the fracture. Also, they found that after hospital discharge, between 34 and 50% of patients were referred to other patient care centers, such as nursing homes, and 24–38% were discharged without the need for formal care, in addition 11–15% were discharged with formal care. The median length of stay was 9.8 [28]. According to the study of Weycker et al., the average length of hospital stay for patients with vertebral fractures in the United States was 5.4 days, and the average hospital cost per patient was $ 11,681 [22].

Among osteoporotic fractures, forearm fractures have almost the lowest cost compared to other fractures. Hospital costs for wrist / forearm fractures in the United States ranged from $ 1,885 to $ 12,136, compared to $ 6,346 to $ 11,236 for vertebral fractures and $ 8,355 to $ 32,195 for hip fractures [21]. In the study of Hernlund et al., the average cost of the hip, vertebral, and forearm fractures one year after fracture in European countries was estimated to be € 13,816, € 3380, and € 989, respectively [10]. In a Chinese study, the average cost of fractures in these common sites during the first year after a fracture was estimated to be $ 4,330, $ 3,409, and $ 1,401, respectively [29]. These costs in our study were estimated at $ 5,381.30, $ 2,980.78, and $ 1,209.30, respectively, which is almost close to the estimated costs in China.

On the basis of the findings of this study and those of other reports, osteoporotic fractures impose a high financial burden on society [10]. There are currently several interventions such as pharmacotherapy and lifestyle modification that are effective and cost-effective in preventing fractures [30, 31]. However, osteoporosis and its related fractures has largely remained globally as an underdiagnose and undertreated health issue [32, 33]. Untreated osteoporosis increases the risk of further fragility fractures [33]. Therefore, eliminating barriers to timely diagnosis and treatment of osteoporosis will lead to fracture prevention.

To the best of our knowledge, this is the first study to estimate the cost within the first year after the common osteoporotic fractures in Iran. However, our study has some limitations. The data were collected from hospitalized patients; therefore, the estimated costs of this study may not be generalizable to non-hospitalized patients. In the case of hip fractures, almost all patients are hospitalized, so our results can be generalized to all of these patients. In the case of vertebral and forearm fractures, according to previous studies, about 35% of vertebral fractures and 25% of forearm fractures require hospitalization [34, 35]. Thus, our estimates for the cost of vertebral and forearm fractures are limited to patients who are admitted to hospital.

Furthermore, in this study, we excluded the patients who died within six months after the fracture. As patients who died after fracture are more severe and could incur higher hospital costs (particularly for hip fracture), excluding them may result in underestimation of the hospitalization costs.

Moreover, outpatient costs in Iran are recorded in multiple databases. Taking this into account that some of the out of pockets costs are not recorded anywhere, so the results of the study might be affected by the recall bias since the outpatient costs were estimated through patients’ interviews.

## Conclusion

Our study showed that common fractures caused by osteoporosis in Iran impose significant costs on patients and society. Due to the increase in life expectancy and the percentage of the elderly population, the incidence and prevalence of osteoporosis and related fractures are increasing that results in higher costs in the future. Implementing cost-effective interventions is necessary to prevent osteoporotic fractures and reduce their associated costs.

## Supplementary Information

**Additional file 1: Table S1.** Methods of measuring direct medical and non-medical costs in this study.

## Data Availability

The datasets used and/or analyzed during the current study are available from the corresponding author on reasonable request.

## Abbreviations

CT: Computed tomography
MRI: Magnetic resonance imaging
BMD: Bone Mineral Density
TUMS: Tehran University of Medical Sciences
