# Supplementary material for: Direct costs of common osteoporotic fractures (Hip, Vertebral and Forearm) in Iran
Source: BMC Musculoskelet Disord. 2021 Jul 31;22:651. doi: 10.1186/s12891-021-04535-8 (PMC8325799; doi:10.1186/s12891-021-04535-8)
Supplement: Supplementary file 1 — Additional file 1: Table S1. Methods of measuring direct medical and non-medical costs in this study. [file 12891_2021_4535_MOESM1_ESM.docx]

**Supplement**

**Table S1: Methods of measuring direct medical and non-medical costs in this study**

| **Cost Item** | | **Method of Evaluation** |
| --- | --- | --- |
| Direct medical cost | Hospitalization costs | These costs were measured by reviewing patients' hospital records. |
|  | Outpatient costs including: Specialist visit, BMD* test, Imaging services**, Rehabilitation services, Laboratory tests, Drugs*** | These costs were measured using a questionnaire through telephone interviews with patients. patients were asked what services they had received over the past six months due to osteoporosis and related fractures, as well as the number of times each service was used.  For this purpose, a list of services including physician visits, imaging services, medications, diagnostic tests, and rehabilitation services was provided, and patients were asked about the use of each service. Then, to calculate the cost of each service used by each patient, the number of services was multiplied by their unit price. |
| Direct non-medical cost | Informal services (including supplements, Walker, cane, and …) | These costs were measured using a questionnaire through telephone interviews with patients. For this purpose, a list of informal services including supplements, Walker, cane, wheelchairs etc. was provided to patients and patients were asked about the use of each service and their paid cost. |
|  | Absenteeism ( by patient and family members) | The patients were asked about the number of absenteeism days by themselves and their family members due to osteoporotic fractures. Then the number of absenteeism days was multiplied by the average income lost per day. |
|  | Informal care | This cost includes the cost of unpaid care provided to dependent patients by a relative such as a spouse, parent, child, etc. To calculate this cost, we first asked patients if they had received informal care and for how long. We then multiplied the number of days each patient received informal care by the official minimum daily wage in Iran. |
|  | Patient time cost | We measured the number of hours patients spent receiving services through interviewing patients, then multiplied the number of hours used by each patient at the official minimum hourly wage in Iran. |
|  | Travel costs | Patients were asked about the number of trips to receive medical care and the average costs of each trip. Then the number of trips by each patient was multiplied by the average costs of each trip. |

*Bone Mineral Density; ** (MRI, CT-SCAN); *** (osteoporosis drug and painkillers)
